# Exploring the Characteristics of Carbon Structures Obtained from LignoBoost Lignin

**DOI:** 10.3390/polym17091221

**Published:** 2025-04-29

**Authors:** Adina Coroabă, Irina Apostol, Ioan Andrei Dascălu, Adrian Bele, Narcisa Laura Marangoci, Florica Doroftei, Cristina Mariana Uritu, Iuliana Spiridon

**Affiliations:** 1 “Petru Poni” Institute of Macromolecular Chemistry, Grigore Ghica Vodă 41 A, 700487 Iași, Romania; adina.coroaba@icmpp.ro (A.C.); apostol.irina@icmpp.ro (I.A.); idascalu@icmpp.ro (I.A.D.); bele.adrian@icmpp.ro (A.B.); nmarangoci@icmpp.ro (N.L.M.); florica.doroftei@icmpp.ro (F.D.); 2 Center of Advanced Research in Bionanoconjugates and Biopolymers, “Petru Poni” Institute of Macromolecular Chemistry, 700487 Iași, Romania; cristina-mariana.uritu@umfiasi.ro; 3 Advanced Center for Research and Development in Experimental Medicine “Prof. Ostin C. Mungiu”, “Grigore T. Popa” University of Medicine and Pharmacy, 700115 Iași, Romania

**Keywords:** lignin, carbon structures, hydrothermal treatment, carbon dot, fluorescence

## Abstract

In the present study, carbon structures from LignoBoost lignin were synthetized using HNO_3_/H_2_SO_4_ one-pot hydrothermal treatment, followed by a thermal treatment. The obtained compounds were characterized using different techniques, such as FTIR, DVS, DLS, XRD, fluorescence imaging and STEM. The formed LCMs presented graphitized structure with quasi-spherical shapes. All obtained materials presented negative values of zeta potential due to the charge from the hydroxyl and carboxyl groups, as confirmed by XPS analysis. All the data obtained sustained the heterogeneous composition of the lignin-based carbon materials, which arise from the complex structure of lignin. Fluorescence imaging demonstrated the potential of the materials as optical imaging agents.

## 1. Introduction

Lignin is a biomass component with an aromatic structure. Its structure varies depending on various factors, including the relative percentages of the syringyl, guaiacyl and p-hydroxyphenyl as primary monomeric units (source of the lignin), delignification process and separation methods [1]. Lignin represents a by-product of papermaking or biorefinery industries, and it could become an important source of materials for high value-added applications. Currently, most lignin is burned to generate heat and electricity [2]. The materials based on lignin or its derivatives are usually environmentally friendly and have a relatively low cost, having applications in environmental remediation, energy storage and other fields [3]. It seems that in many potential applications of lignin, lignin is a valuable candidate for carbon precursors [4,5,6,7].

Considering that carbon materials are derived from petroleum-based chemicals and the pressure of environmental legislation, the interest in lignin valorization through the development of carbon materials highly increased. Unfortunately, the production of crystalline carbon structures is challenging because of the poor capacity of lignin to be graphitized. The literature data mention that the high activity of alkali lignin recommends it as the precursor of carbon materials [8]. The complex structure of lignin, rich in covalent bonds such as C–C and C–O, prevents its depolymerization. This is why acid hydrolysis treatment is performed to facilitate the hydrothermal treatment of lignin.

The preparation of lignin carbon dots (CDs) via a hydrothermal reaction has attracted the interest of the research community. Thus, some authors [9] obtained lignin-derived CDs for bioimaging and sensing, while Lin et al. [10] synthetized CDs from enzymatic hydrolysis lignin. They evidenced the presence of oxygen-, nitrogen- and sulfur-functional groups, which confer fluorescence properties to the synthetized CDs. Other authors [11,12] reported the obtainment of alkali lignin-based carbon dots with bright fluorescence and long-term photostability. Zhou et al. [13] have used sulfuric acid, tungstic acid, phosphoric acid and boric acid to synthesize polychromatic fluorescent lignin carbon dots with advanced functionalities.

Despite the high interest in CDs, there are some gaps related to their obtainment and cost-effective synthesis methods and their applications. Their potential applications are gaining momentum, and more studies are being performed on this topic.

The purpose of this work was to investigate the influence of treatment of Lignoboost lignin on the characteristics of the obtained lignin-based carbon materials (LCMs). This approach represents a sustainable way to valorize Lignoboost lignin into valuable materials, thus contributing to the reduction in waste and reliance on fossil-based materials. Our results showed that the applied treatments, carried out under milder conditions as compared to the literature data, resulted in different lignin-based carbon materials with different sizes and optical emission properties. Using fluorescence imaging, the potential application of all lignin-based carbon materials as optical imaging agents was demonstrated, a topic of significant interest for the medical community.

## 2. Materials and Methods

### 2.1. Reagents

Lignoboost (LB) softwood lignin was obtained by Södra Cell, Väröbacka, Sweden, using the Lignoboost process, which involves acid precipitation (with CO_2_) of the black liquor from the kraft mills, dewatering and conditioning [14]. HNO_3_, H_2_SO_4_, acetone and ethanol, as well as ethylenediamine (EDA) were purchased from Sigma-Aldrich (St. Louis, MO, USA).

### 2.2. Obtaining of LCM

A total of 2 g of Lignoboost lignin, dispersed into 10 mL water, was mixed with 3 mL of HNO_3_ (65% wt)/H_2_SO_4_, (96% wt). The mix was heated at 90 °C with continuous stirring at a rate of 250 rpm. It was cooled to room temperature after 4 h, washed and sonicated during 4 h. Then, a volume of 30 mL solvent (acetone or ethanol) was added to the washed reaction product. The mixture was subjected to thermal treatment carried out at 95 °C, 50 mbar, for 8 h (Table 1). Once the samples had cooled to ambient temperature, the suspension was vacuum filtered through a 0.22 μm poly(vinylidene fluoride) (PVDF) filter to remove unreacted lignin. The filtrate was then dialyzed through a dialysis membrane (1000 Da) for 4 days. The purified LCMs were dried in an oven at 60 °C and stored for further characterization.

To the samples treated with H_2_SO_4_, 0.6 mL of ethylenediamine (EDA) was added as a passivating agent before sonication.

### 2.3. Characterization of LCMs

#### 2.3.1. Fourier Transform Infrared Spectroscopy (FTIR)

The functional groups of each sample were confirmed using an FTIR instrument (Vertex 70 from Brüker (Billerica, MA, USA), outfitted with an ATR device (ZnSe crystal) set at a 45-degree angle of incidence) in the wavenumber range of 4000–400 cm^−1^. The measurements employed an average of 64 scans with a spectral resolution of 2 cm^−1^.

#### 2.3.2. X-Ray Photoelectron Spectrometry (XPS)

X-ray photoelectron spectroscopy (XPS) analysis was performed using a 5000 Versa Probe spectrometer (Φ ULVAC-PHI, Physical Electronics Inc., Chanhassen, MN, USA), equipped with a monochromatic Al Kα X-ray source (hυ = 1.486 keV). The binding energy calibration was conducted using the C 1s peak at 284.6 eV as a reference. Peak deconvolution and data analysis were carried out using CasaXPS software (version 2.3.26PR1.0).

#### 2.3.3. Dynamic Light Scattering (DLS) and Zeta Potential

The particle size distribution was analyzed by dynamic light scattering using a Malvern Panalytical Zeta-sizer Advance Pro Red instrument (Malvern Panalytical Ltd., Malvern, UK), at a constant temperature of 25 °C. The samples were dispersed in deionized water (pH 6.9) with a concentration of 1 mg/mL. Solutions were sonicated for proper distribution, centrifugated at 5000 rpm for 10 min and then analyzed. The reported results represent average of three samples ± standard deviation.

#### 2.3.4. Dynamic Vapor Sorption (DVS)

A DVS analyzer from Hiden Analytical, Warrington, UK was used to measure the water vapor uptake in the dynamic regime, as a function of an increase in humidity. The thermal studies were performed as a function of humidity (0–95%) in the temperature range from 5 °C to 85 °C, with an accuracy of ±1% for 0–90% RH and ±2% for 90–95% RH. The adsorption isotherms allow us to evaluate the BET specific surface area, using the Brunauer–Emmett–Teller (BET) equation. The reported results represent average of three samples ± standard deviation.

#### 2.3.5. X-Ray Diffraction

The X-ray diffraction patterns of the samples were measured as Θ–2Θ scans with a Rigaku SmartLab X-ray diffractometer in Bragg–Bretano geometry using a Cu anode (X-ray wavelength of 1.5406 Å), in the angular range 5–60° (2θ), with a scanning step of 0.02° and a recording rate of 3°/min.

#### 2.3.6. Fluorescence Properties

Steady-state fluorescence was measured using a Horiba Fluoromax 4 spectrofluorometer (Horiba Ltd., Kyoto, Japan). The measurement parameters, including the entrance and exit slits, as well as the integration time, remained constant across all samples. The FluorEssence software (ver. 3.5.1.20) was used for data acquisition and subsequent data representation. For this investigation, water was used as the solvent.

#### 2.3.7. Fluorescence Imaging

Fluorescence imaging of the compounds was conducted using the AMI HTX system from Spectral Instruments Imaging (Tucson, AZ, USA), designed for small animals. This equipment is capable of acquiring images in fluorescence, bioluminescence and X-ray modes. It features a high-performance CCD camera cooled to −90 °C, ensuring exceptional sensitivity and minimal background noise during image acquisition. The system enables the detection and quantification of optical signals corresponding to the selected imaging mode. It can be configured to operate at 10 specific excitation wavelengths ranging from 430 to 745 nm, with emission detection between 530 and 790 nm. Data analysis was carried out using Aura software, which facilitates quantitative image processing. The samples were prepared by diluting the stock solutions of 10 mg/mL in ultrapure water to obtain the following concentrations: 0.5, 0.75, 1, 1.25, 1.5, 2, 2.5 and mg/mL. The serial concentrations of each compound were placed in the wells of a plate for cell culture (with 96 wells) by diluting in both ultrapure water and 1% agarose gel. The fluorescence emission was measured at excitation and emission wavelengths of 430 and 530 nm, respectively, while the excitation power was set to 15 and 60%.

#### 2.3.8. Scanning Transmission Electron Microscopy (STEM)

The morphology of the synthesized samples was analyzed in STEM Mode with a Verios G4 UC Scanning electron microscope (Thermo Scientific, Brno, Czech Republic) equipped with an Energy Dispersive X-ray spectroscopy analyzer (Octane Elect Super SDD detector, USA). The STEM studies were performed using the STEM 3+ detector (Bright-Field Mode) at accelerating voltage of 25 kV. For STEM in SEM analysis, the samples were dispersed in water, ultrasonicated and then they were placed on carbon-coated copper grids with 300-mesh size and dried in an oven until the solvent was removed.

## 3. Results

### 3.1. FTIR Spectra

**Figure 1:**
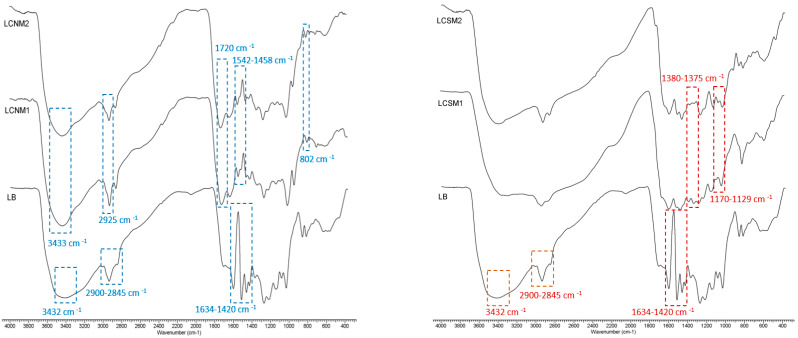
FTIR spectra of LCSMs obtained in the presence of HNO_3_ (**left**) and H_2_SO_4_ (**right**).

FTIR spectra of lignin and LCMs are presented in Figure 1. In lignin, aromatic skeletal vibration related bands occurred at 1598 cm^−1^, 1510 cm^−1^ and 1420 cm^−1^ [15], while the stretching vibration peaks of the –CH_3_ and –CH_2_ are around 2900 cm^−1^. Also, lignin-specific –CH_2_– and tertiary C–H groups presented symmetric stretching vibrations at 2845 cm^−1^.The stretching vibration peaks of –OH are observed at 3432 cm^−1^. The peak at 756 cm^−1^ (bending vibration of C–S bond) confirms the presence of sulfur in LignoBoost lignin.

The peak at 1425 cm^−1^ specific to –C–C– stretching (aromatic) disappeared, and new peaks occurred in the spectra of lignin-based carbon materials. The characteristic band for C=C vibration at 1634 cm^−1^ indicates the presence of sp^2^ hybridized carbon atoms with hydroxyl, carbonyl and carboxylic functional groups [16]. The spectra of LCNMs evidenced a broad absorption at 2925 cm^−1^ caused by C–H stretching vibrations and the absorption peaks at 1458 can be attributed to the stretching vibrations of the C=O bond, while a new peak at 1542 cm^−1^ is ascribed to the C=C or C–N bonds, respectively [17]. The peak occurring at 1720 cm^−1^ is assigned to the non-conjugated C=O stretching vibration, correlated with the oxidation of HNO_3_, demonstrating that LCMs inherit the aromatic skeletons from LB and possess more π-conjugated structures as compared to LB [18], which explains the fluorescence properties of the synthetized materials. The peak occurring at 802 cm^−1^ was associated with the N–H out-of-plane bending vibration [19], while the peak at 1315 cm^−1^ can be assigned to graphitic nitrogen, in agreement with XPS results. In the spectra of LCSMs, the peaks that occurred at 1152 and 628 cm^−1^ are attributed to the stretching vibrations of C–S [20], confirming the existence of sulfur-based functional groups in lignin-based carbon materials structure.

### 3.2. X-Ray Photoelectron Spectrometry (XPS)

**Figure 2:**
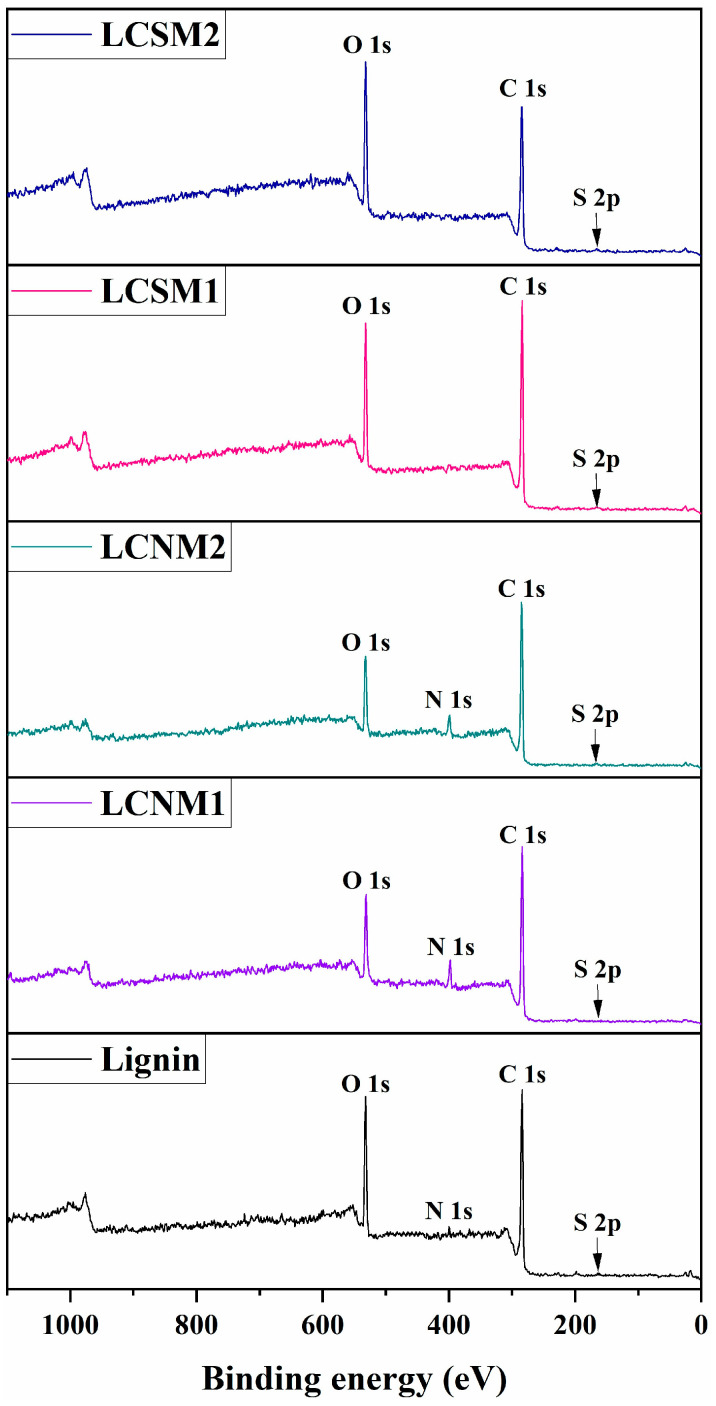
XPS wide scan spectra of the investigated samples.

X-ray photoelectron spectroscopy (XPS) was used to investigate the surface composition of the samples. The analysis revealed differences in the elemental composition of the samples examined. Table 2 presents the weight percentages of carbon (C 1s), oxygen (O 1s), sulfur (S 2p) and nitrogen (N 1s) derived from the XPS wide scan spectra of each sample (Figure 2), facilitating a comparative analysis of elemental distributions among various sample types.

The LB lignin spectrum exhibited three distinct peaks corresponding to C 1s, O 1s and S 2p, with weight concentrations of 75.29%, 22.68% and 2.03%, respectively. The LCMs samples displayed varying compositions attributed to different synthesis methods and treatment conditions, as reported in the literature [21,22]. In the case of LCNMs samples, an additional peak specific to N 1s is observed, exhibiting varying mass concentrations due to the HNO_3_ treatment conditions (8.02% for LCNM1 and 6.08% for LCNM2). In contrast, the LCSMs samples presented an increased content of S 2p as a result of the H_2_SO_4_ treatment (3.88% for LCSM1 and 3.71% for LCSM2).

**Figure 3:**
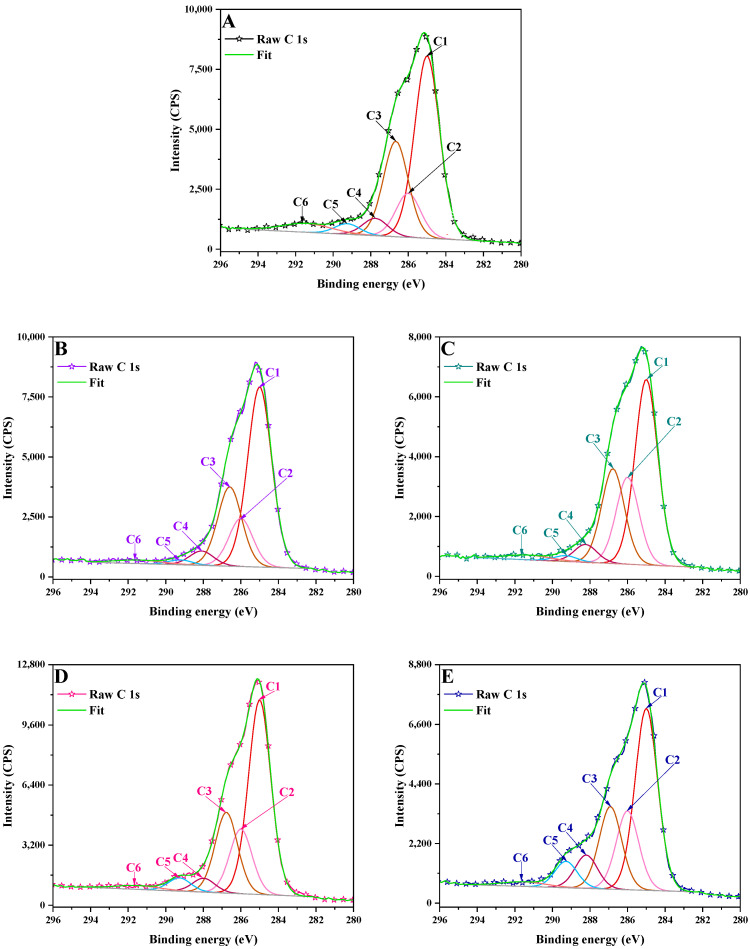
XPS high resolution spectra of C 1s signal in: (**A**) LB, (**B**) LCNM1, (**C**) LCNM2, (**D**) LCSM1, (**E**) LCSM2.

The deconvoluted spectra of the C 1s (Table 3, Figure 3) revealed that all the materials presented six distinct peaks assigned to the C=C/C–C/C–H, C–SO/C=N, C–OH/C–N, C=O/C–O–C, O–C=O bond and π–π* satellites.

The evaluation of chemical states revealed that LCNM1 and LCSM1 presented more carbon in sp^3^ C–C/sp^2^ C=C hybridization, as compared to lignin. The effects of oxidation and doping by HNO_3_ can introduce C=O and graphitic nitrogen structures into LCMs, thereby increasing the sp^2^ carbon ratio [12].

**Figure 4:**
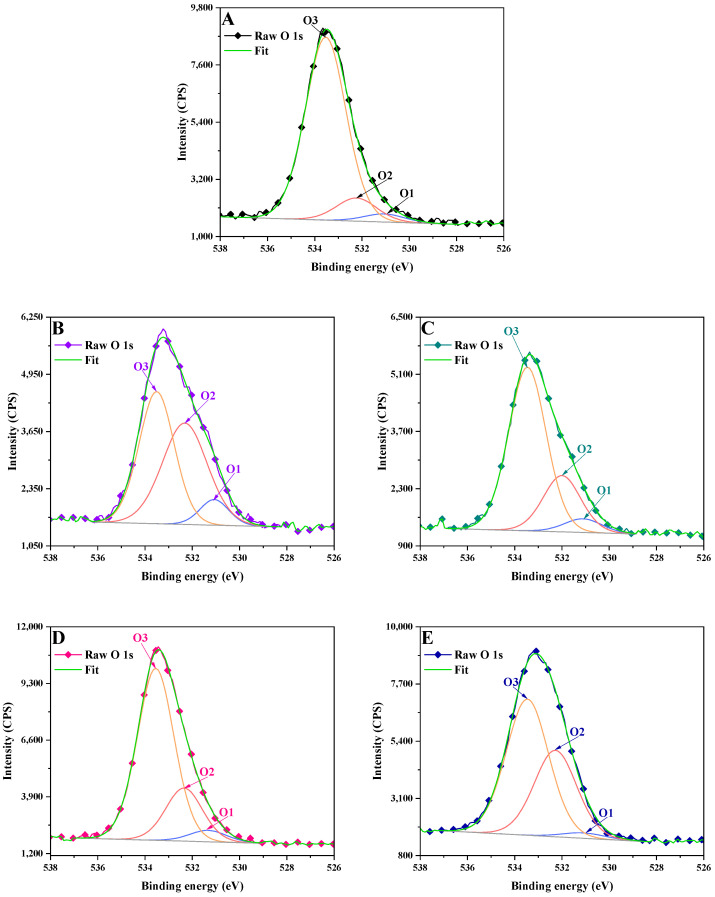
XPS high resolution spectra of O 1 signal in: (**A**) LB, (**B**) LCNM1, (**C**) LCNM2, (**D**) LCSM1, (**E**) LCSM2.

The O 1s peaks were deconvoluted into three peaks, as follows: the –C=O peak at ~531.1 eV, O=C–O–peak at ~532.2 eV and ~533.5 eV [23], corresponding to –C–OH bonds (Table 4, Figure 4).

The high content of C=O bonds illustrates the oxidation of the hydroxyl groups in LignoBoost lignin to aldehyde and quinone structures.

**Figure 5:**
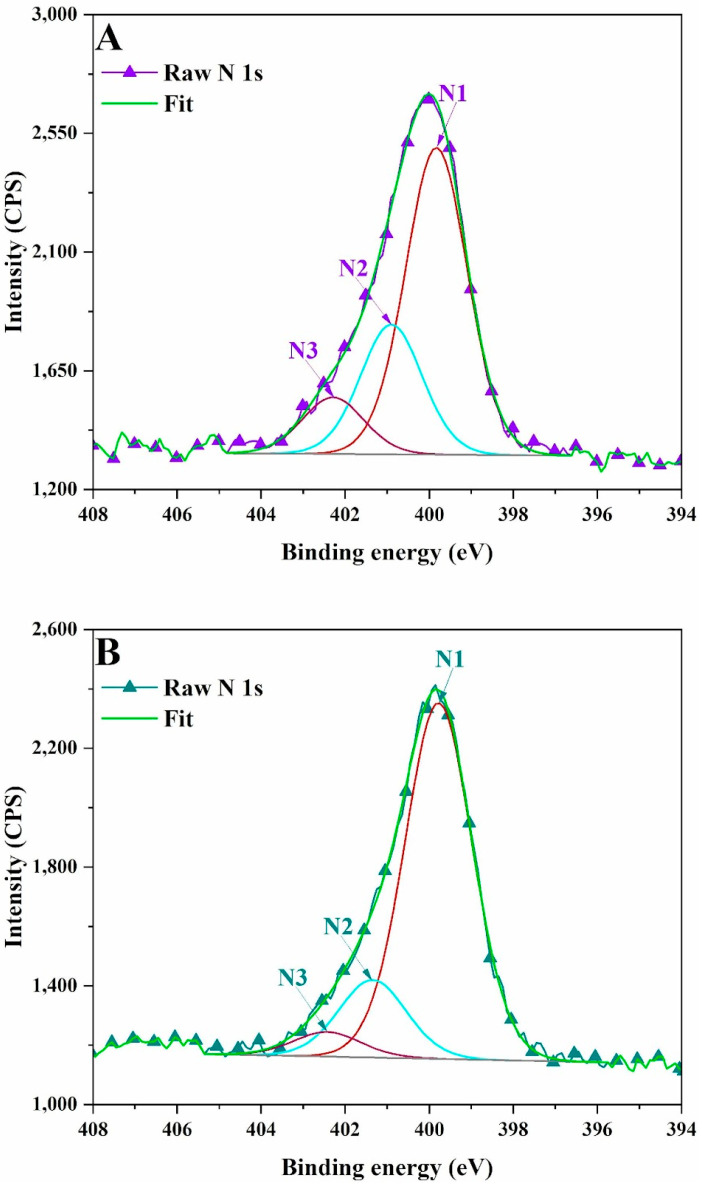
XPS high resolution spectra of N 1s signal in: (**A**) LCNM1, (**B**) LCNM2.

**Figure 6:**
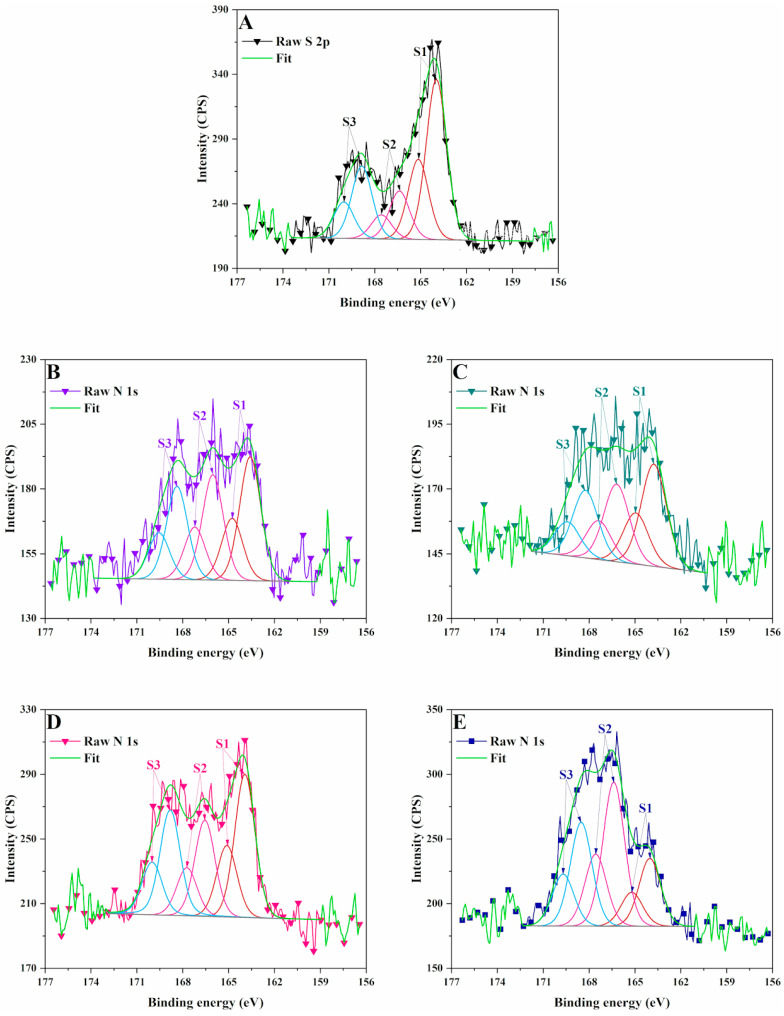
XPS high resolution spectra of S 2p signal in: (**A**) LB, (**B**) LCNM1, (**C**) LCNM2, (**D**) LCSM1, (**E**) LCSM2.

The N 1s peak (Table 5, Figure 5) was divided into three peaks that are attributed to pyridinic N (398.9 eV), graphitic N peak (401.3 eV) and N–H/quaternary-N (402.3 eV) [24]. These nitrogen species in the carbonic structures surface come from the reactions between nitric acid and the hydroxyl groups from lignin at high temperature, accompanied by a reduction in the oxygen content. LCSM samples synthetized using sulfuric acid presented a higher content of sulfur as compared with lignin (Table 5, Figure 6). The XPS spectrum of S 2p region presented three peaks corresponding to S 2p3/2, C–S–C and highly oxidized SO_x_ respectively [25]. The presence of S 2p3/2 and S 2p1/2 is attributed to the C-S bonds. –SO_x_ improve electrochemical activity, as well as the wettability of carbon-based materials. The contribution corresponding to –C–S(O)_2_–C– sulfone bridges increased.

Our findings suggest that the synthetized LCMs are enriched in different groups, such as O–H, –COOH and N–H, which can influence their fluorescence properties.

### 3.3. Dynamic Light Scattering

**Figure 7:**
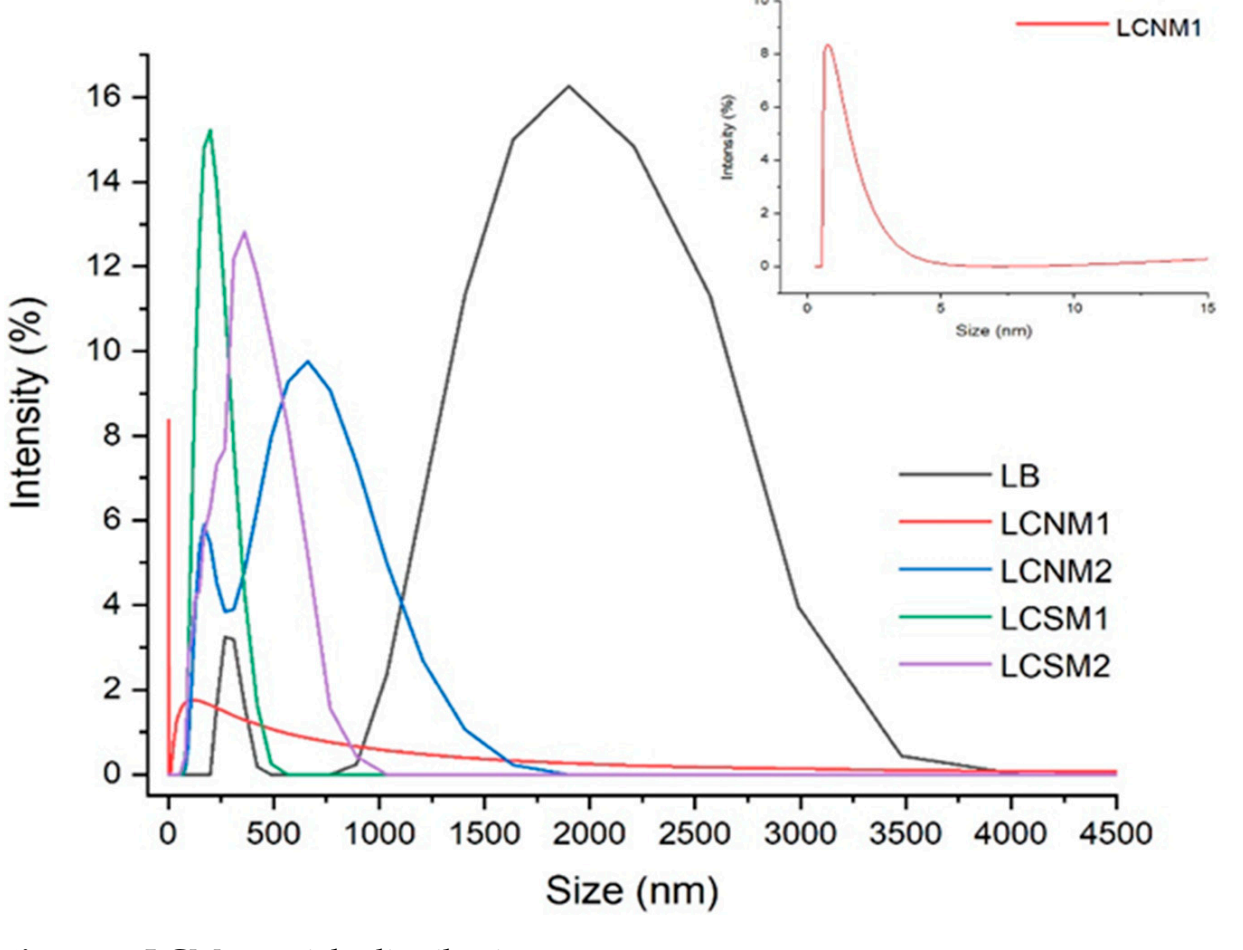
LCMs particle distribution.

DLS evidenced that LCMs particles of various sizes were obtained (Figure 7). Our LCMs exhibited a broad size distribution, indicating the heterogeneous nature of the materials. LCNM1 presented the lowest mass average particle size, as well as number average particle sizes, while the other materials presented higher values of apparent hydrodynamic diameter. This might be caused by the electrostatic attraction interactions between the functional groups present on the surface of the LCSMs.

The zeta potential of LCMs (Table 6) was also investigated. The surface charge gives information related to the strength of the electrostatic attraction between the like-charged particles in a dispersed system [26]. Lignin presented a zeta potential of −31.06 mV, which can be due to the negative charges of the phenol groups, and partially to the adsorption of hydroxyl ions on its hydrophobic surface [27]. All obtained materials presented negative values due to the charge from the hydroxyl and carboxyl groups [28], as confirmed by XPS studies. Similar results were reported by Aldakhil et al. [29]. Thus, the zeta potentials of materials decreased in the order of −40.79 (LCNM1) > −37.11 (LCSM1) > −32.41 (LCNM2) > −31.06 (Lignin) > −29.84 (LCSM2), suggesting reduced dispersion stability and a tendency toward aggregation. LCNM1 presented the highest zeta potential, indicating strong electrostatic repulsion between the particles, which assures very good dispersion and stability. This might be correlated to the content of –COOH that holds the potential of deprotonation [30]. This is well correlated with XPS data (Table 4).

The lower value registered for LCSM2 suggests the formation of clusters, which means that there are numerous dipolar interactions causing a reduction in the charge transfer between the particles of material and solvent molecules, according to Zaini et al. [31]. STEM images confirm this supposition.

### 3.4. Dynamic Vapor Sorption

**Figure 8:**
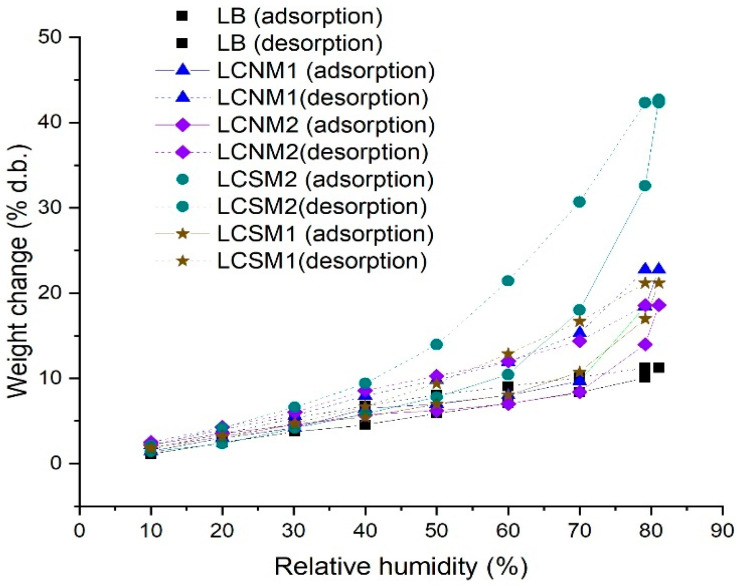
Plotted DVS data of the LCMs.

The DVS method was used to assess the sorption behaviors of LCMs under consideration. The data from Table 7 and Figure 8 evidence that both BET surface area and sorption capacity depend on the synthesis methods.

It is well known that the specific surface area is related to the amount of the adsorbed moisture on the first monolayer (when it is completely covered). According to Huo et al. [32] during carbonization, lower molecular weight lignin precursors produce fewer defects and higher specific surface areas.

Our data show that the solubilization in acetone, followed by the thermal treatment in autoclave for 8 h, resulted in the development of a more porous structure (LCSM1 and LCNM1, respectively) and a high value of BET surface area. Materials obtained by this procedure presented the same water sorption capacity trend. Materials solubilized in ethanol before thermal treatment recorded a decrease in BET surface area, which could be related to the amount of closed pores during the thermal treatment. The high amount of the hydroxyl/carboxyl groups (see XPS data) present on the BET surface area of LCSM2 contributed to their easier dispersal in water [33].

### 3.5. X-Ray Diffraction

**Figure 9:**
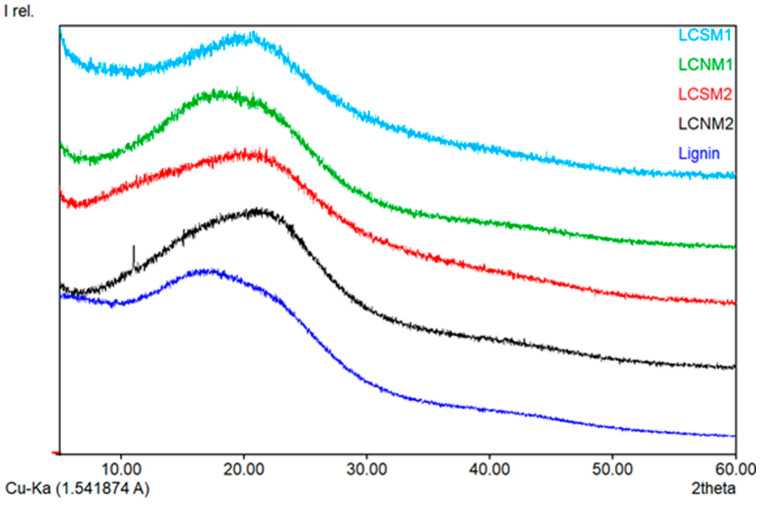
XRD patterns of the LCMs samples.

The XRD spectrum of LignoBoost lignin proves its amorphous nature, attributed to the presence of numerous aromatic rings within the molecule and the resulting polymeric chains, distributed in a disordered arrangement. Following the lignin transformation into LCMs, the broad amorphous peak observed for lignin (Figure 9) shifted towards higher 2θ values. The poorly ordered structure of LCMs could be explained by the significant steric hindrance of mono-benzene rings within the amorphous structure of lignin, which affects the interaction forces between the benzene units.

In LCNM1, the diffraction peak was gradually displaced to 23°, suggesting that the amorphous lignin was transformed into materials with partial graphite structure having more nitrogen atoms (which have the atomic radius smaller than the carbon atoms) introduced into the surface of the materials as a result of HNO_3_ use during the synthesis [34]. All LCSM structures presented a (002) peak, belonging to the graphite structure. The interlayer spacing values were 4.75 Å (LCNM1), 4.74 Å (LCNM2), 4.43 Å (LCSM1) and 4.28 Å (LCSM2), larger than that of bulk graphite (3.3 Å), indicating poor crystallization [35]. The low crystallinity of lignin carbon dots was reported by other authors as well, who attributed it to the oxygen containing groups present on the material surface, enhancing the interlayer distance [36]. Due to their heterogeneous structure, lignins often show unexpected behavior. When hardwood, softwood and non-wood lignins were mixed with a eutectic salt mixture to obtain carbon materials [37], these presented similar interlayer spacing in the crystals, but the crystal size and the degree of crystallinity varied, all ordered structures being randomly oriented. The C-O-C or C–C bonds are cleaved during acid hydrolysis. Under the acidic conditions of the hydrothermal reaction, H^+^ attacks the C_α_ of the phenylpropanoid units from lignin to form a carbocation [38]. The lignin carbocation structural unit mainly undergoes two reactions. One is fragmentation by acidolysis of β-O-4 structure, forming a carbonyl group and a new phenolic end group, and then the lignin macromolecule is cleaved into small molecules. Demethoxylation, dissociation of aliphatic side chains, cleavage of aryl ether linkages and condensation reactions will break the weaker bonds between the lignin subunits and remove the methoxy groups. The aromatic C_5_ or C_6_ of another lignin structural units can polymerize to form carbon–carbon bonds. These two reactions occur at the same time. With the increase in the number of small lignin molecules, the polymerization reaction becomes the main reaction and LCMs are formed. The reactive lignin fragments with short sidechain might be deoxygenated and restructured to generate the small fraction during the solvothermal reaction under the aromatic refusion via the prompt sp^2^ hybridization and π-π stacking [39]. According to XPS data, more functional groups containing oxygen, nitrogen and sulfur were introduced, resulting in more active sites on the surface of LCMs. Lin et al. [10] reported that the structures having oxygen-, nitrogen- and sulfur-functional groups on their surface, presented good fluorescence properties. Due to the differential lignin fragments, the manipulation of acid-reagent engineering triggers the variations in physicochemical properties of LCDs that jointly direct their multicolor emission to broaden the sensing applications [40]. Our synthetized LCMs presented good fluoresce properties and significant differences between them were recorded, as a function of the acid used in the synthesis process. Moreover, having in mind the discrepancy in lignin segments, the solvent polarity used in the solvolysis reaction resulted in the alterable properties of LCMs that differ in fluorescence properties, fact that was also reported by Zhu et al. [41].

It was reported that ethylenediamine (EDA), used for surface passivation, introduced the N-atom possibly into the carbon nuclear lattice of the graphitic carbon of lignin [42,43].

Park et al. [44] reported the presence of sulfur- and nitrogen-related functional groups in lignin carbon dots synthetized from lignin and EDA.

Our XPS data did not confirm the presence of N on the LCSMs surface, probably due to the mid synthesis conditions.

### 3.6. Fluorescence Properties

**Figure 10:**
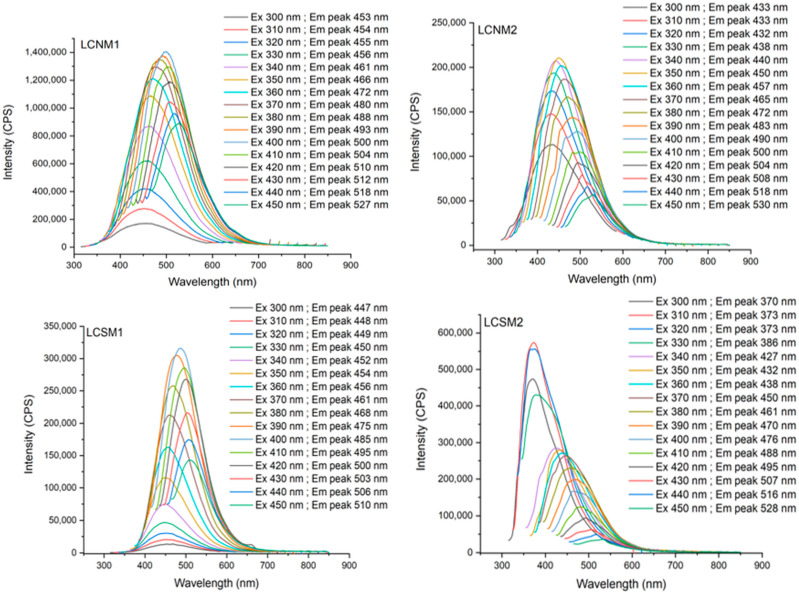
Fluorescence emission spectra of LCMs dispersed in water at 1 mg mL^−1^ concentration under different excitation wavelengths.

The fluorescence properties are influenced by the carbon core eigenstates, as well as by the surface defect states [27]. Fluorescence emission properties of the synthetized LCMs were investigated at different excitation wavelengths. The findings demonstrated that the overall fluorescence emissions of all LCMs were in the blue-green domain. According to Figure 10, the maximum emission spectra gradually shifted in the direction of the long wavelength when the excitation wavelength ranged from 300 nm to 450 nm. This suggests an excitation-dependent fluorescence emission behavior similar to that observed in other carbon dots derived from lignocellulosic biomass reported in the literature [45]. The observed red shift suggests that the materials exhibit multiple emissive states, potentially resulting from the heterogeneous surface functionalities identified in FTIR analysis and/or the broad particle size distributions revealed by DLS measurements. The materials presented different excitation–emission behavior, most probably influenced by the acidic treatment (HNO_3_ or H_2_SO_4_) of the Lignoboost lignin and by the solvents (acetone and ethanol) used in the preparation steps. Some authors reported that the presence of electron-withdrawing groups, such as nitro groups, causes a decrease in the fluorescence intensity [46]. Figure 10 indicates that LCNM1 exhibited the highest fluorescence intensity among all samples, while LCSM1 showed decreased intensity. LCNM2 had weaker fluorescence as compared to LCNM1, characterized by lower overall intensity, but a similar trend in excitation-dependent emission. LCSM2 also demonstrated fluorescence properties; however, its intensity is considerably reduced. It is noteworthy that LCNM2 exhibited greater intensity values in the 300–340 nm excitation range compared to the other three samples, most probably due to the fact that the sulfur atoms modified the O-states [47].

Although all the samples presented heterogeneous nature, possibly due to variations in particle size and surface functionalities, it can be observed that LCNMs samples have a broader and a more pronounced emission profile as compared to LCSMs samples, suggesting differences in their structural or chemical composition (confirmed by FTIR and XPS analysis), which can influence their fluorescence properties. Also, LCNMs samples are displaying peaks in the longer wavelength region (higher red-shift) compared to LCSMs. The more extensive π-electron system or the higher graphitic nitrogen content caused a narrower energy gap in LCNMs samples, shifting their fluorescence emission to the red region, as Bao et al. reported [48].

**Figure 11:**
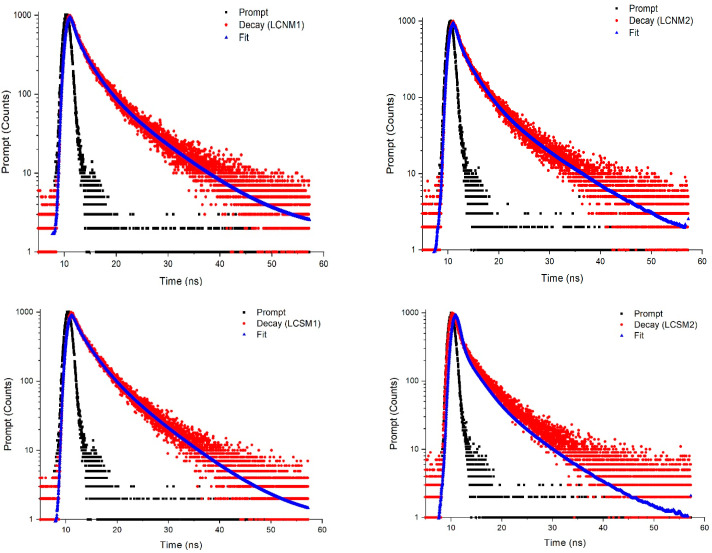
Time-resolved photoluminescence spectra of LCDs at λ_ex_ of 370 nm.

The time-resolved emission spectra of the LCMs solutions, fitted with a multi-exponential decay model (Figure 11), revealed an average lifetime between 6.19 and 7.49 ns (Table 8), consistent with other research on fluorescent carbon nanostructures [49,50]. These lifetime values suggest that the luminescence of the LCMs is caused by their surface state, with defects potentially leading to the compounding of electrons and holes, with an impact on the release energy in the form of photoemission.

The lowest τ1 component associated with the eigenstates was recorded for LCNM2, while the lowest τ2 component associated with the surface defect states was registered for LCSM2. Nguyen et al. [51] reported that the long lifetime of materials is correlated with the surface functional groups.

The solvent used during the synthesis of lignin-derived carbon materials (acetone and ethanol) also affects the fluorescence lifetime, suggesting that the contribution of the carbon core to the emission of LCNMs gradually decreases, while the surface states, such as functional groups, become increasingly important. Therefore, it can be concluded that surface states, rather than the carbon core, play a dominant role in the photoluminescence, where the excited state energy is captured, leading to stronger emission. A similar behavior was observed by Song et al. [52] for carbon dots doped with N.

### 3.7. Fluorescence Imaging

**Figure 12:**
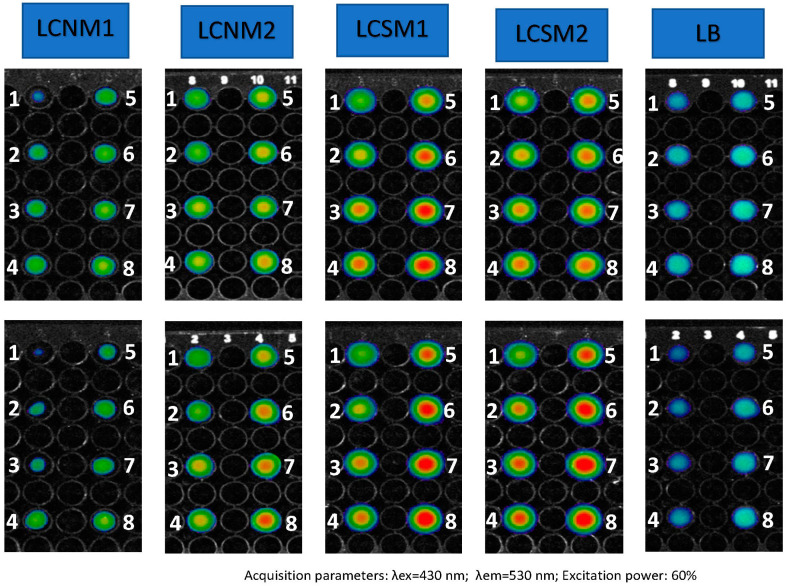
Fluorescence images of the materials, dispersed in 1% agarose gel (**upper line**) and ultrapure water (**bottom line**), at serial dilutions from 0.5 to 3 mg/mL (1–8).

**Figure 13:**
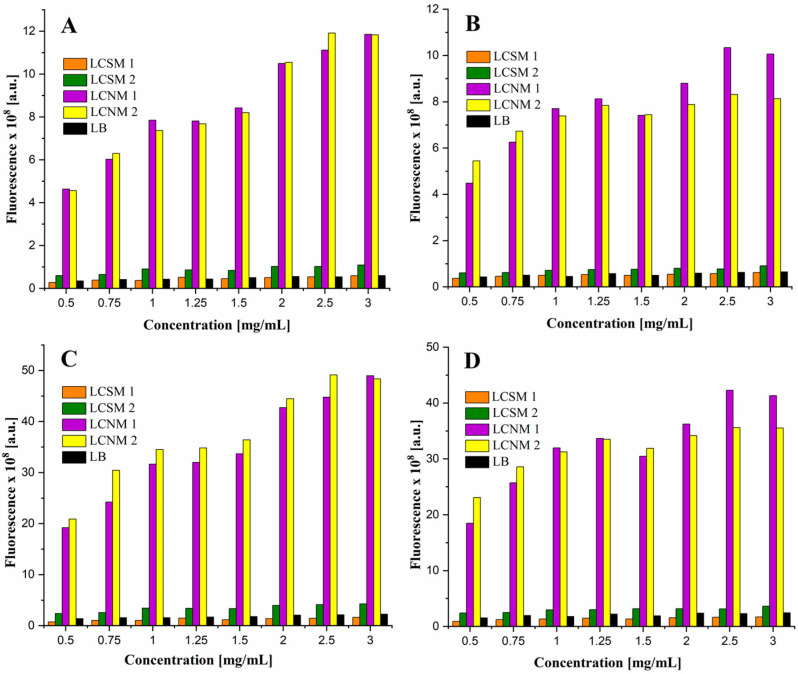
Fluorescence intensity versus samples concentration ((**A**) ultrapure water, PE = 15%; (**B**) agarose gel, PE = 15%; (**C**) ultrapure water, PE = 60%; (**D**) agarose gel, PE = 60%).

The excitation and emission wavelengths were chosen based on the fluorescence spectra of the compounds, and the closest predefined values were 430 and 530 nm, respectively. The agarose gel had the role to keep a better dispersion of the particles in the volume of the samples, but it also mimicked cell cultures and biologic medium [53], helping to assess the potential influence of the tissues on fluorescence emission. The fluorescence images obtained for 60% excitation power are shown in Figure 12. It is evident that the samples in agarose show lower optical signals compared to those in water, but the dispersion is much more uniform throughout the volume of the wells, which is supported by Figure 13. All the samples proved interesting behavior as fluorescent agents. Although samples LCSM1 and LCSM2 exhibited significantly lower fluorescence as compared to samples LCNM1 and LCNM2, they still remain detectable on the well plate at all concentrations when an excitation power of 60% is applied. The fluorescence reduction by a factor of 1.1–1.4 upon agarose gel embedding suggests that some of the emitted fluorescence is absorbed by the surrounding tissues. Therefore, these compounds are more effective as optical imaging agents for visualizing surface structures rather than deeper structures, such as skin tumors, superficial blood vessels and similar features of the organisms. These findings align with recent research in bioimaging, indicating that compounds emitting in the UV-Vis spectrum are more appropriate for surface proximity, whereas NIR-I (650 to 950 nm) and NIR-II (1000 to 1400 nm) enhance image quality and tissue penetration, thereby expanding the applications of this technique for disease diagnosis and treatment monitoring [54].

### 3.8. STEM Images

**Figure 14:**
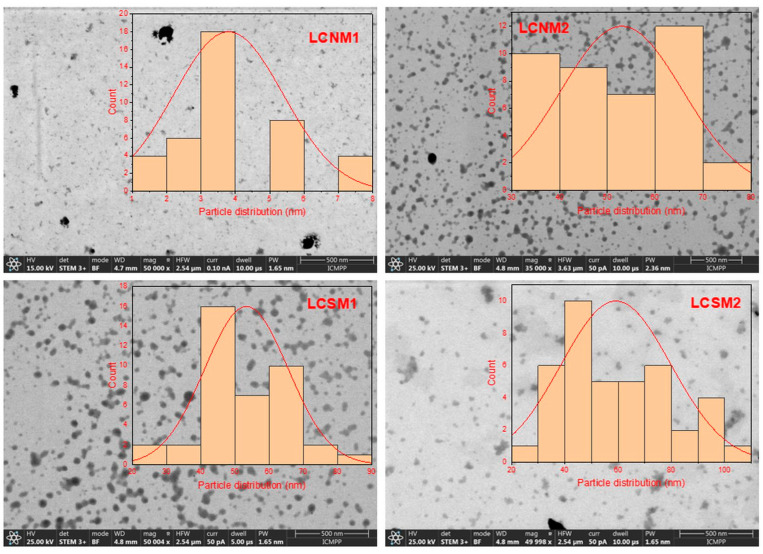
STEM images of LCMs.

STEM images (Figure 14) show that most LCMs particles are relatively uniformly dispersed, exhibiting quasi-spherical shapes. These particles were randomly measured using Image J 1.53e software. It was evidenced that the synthesized LCMs were well dispersed and quasi-spherical particles with broad size distribution, varying between 1.1 nm and 7.19 nm for LCNM1, while for other LCMs varied between 24.9 and 82.7 (LCSM1), 29.6 and 107.8 (LCNM2) and 37.035 and 78.8 (LCNM2, respectively (Figure 14, inset). These are lower than the hydrodynamic diameter of a particle, assessed by the DLS technique. According to STEM images, except LCNM1 sample, the other LCMs tend to form aggregates, in agreement with DLS data. This phenomenon is supported by the theory of “blackened carbon”, the differences between samples being attributed to the difference in zeta potential [55]. Ayilliath et al. [56] explained that the interactions between functional groups facilitate the formation of particle aggregates.

## 4. Conclusions

A series of lignin-based carbon materials were prepared by HNO_3_/H_2_SO_4_-assisted one-pot hydrothermal preparation, followed by thermal treatment. It was found that nitric acid was more efficient to promote lignin depolymerization and re-combination of lignin fragments to form carbon dots (LCNM1). The synthetized LCMs presented graphitized structure with quasi-spherical shapes. All obtained materials presented negative values of zeta potential due to the charge from the hydroxyl and carboxyl groups, as confirmed by XPS analysis. The BET surface area, as well as the water sorption capacity of the materials depended on the synthesis methods.

All LCMs demonstrated excitation-dependent fluorescence emission behavior, a characteristic commonly observed in carbon dots derived from lignocellulosic biomass, as reported in previous studies. The fluorescence data sustained the heterogeneous composition of the LCMs, which can arise from the complex structure of lignin. Fluorescence imaging demonstrated a behavior good enough to inspire optimism for the potential applications as optical imaging agents in medicine.

This work provides solutions for the conversion of lignin sourced from biomass waste into high-value products with a low carbon footprint. However, the botanical and technical heterogeneity of lignin requires more studies to be carried out in order to assure the fluorescence tunability for further advanced applications. 

## Figures and Tables

**Table 1:** Conditions for the obtainment of LCMs.

Code	Acid	Solvent	Thermal Treatment
LCNM1	HNO_3_	Acetone	Autoclave, 95 °C, 50 mbar, 8 h
LCNM2	HNO_3_	Ethanol	Autoclave, 95 °C, 50 mbar, 8 h
LCSM1	H_2_SO_4_	Acetone	Autoclave, 95 °C, 50 mbar, 8 h
LCSM2	H_2_SO_4_	Ethanol	Autoclave, 95 °C, 50 mbar, 8 h

**Table 2:** Surface composition of the samples obtained from XPS analysis.

Sample	Element (wt%)			
	**C 1s**	**O 1s**	**S 2p**	**N 1s**
LB	75.29	22.68	2.03	-
LCNM1	72.80	18.77	0.42	8.02
LCNM2	76.31	17.34	0.28	6.08
LCSM1	73.77	22.35	3.88	-
LCSM2	68.43	27.87	3.71	-

**Table 3:** The percentages of carbon-containing functional groups of materials.

Sample	C 1s											
	C1		C2		C3		C4		C5		C6	
	C=C/C–C/C–H		C–SO/C=N		C–OH/C–N		C=O/C–O–C		O–C=O		π-π*	
	At. Conc. (%)	BE (eV)	At. Conc. (%)	BE (eV)	At. Conc. (%)	BE (eV)	At. Conc. (%)	BE (eV)	At. Conc. (%)	BE (eV)	At. Conc. (%)	BE (eV)
LB	49.59	285	11.98	286	25.97	286.7	4.72	287.8	2.90	289.3	4.83	291.4
LCNM1	53.96	285	14.42	286	23.76	286.6	4.29	288.1	1.45	289.2	2.10	291.5
LCNM2	46.38	285	21.71	286	23.65	286.8	4.46	288.2	1.45	289.3	2.36	291.3
LCSM1	51.93	285	17.24	286	21.56	286.8	3.70	288	3.55	289.3	2.02	291.5
LCSM2	44.10	285	19.13	286	20.05	286.9	8.08	288.2	6.41	289.3	2.23	291.5

**Table 4:** The percentages of oxygen-containing functional groups of materials.

Sample	O 1s					
	O1		O2		O3	
	–C=O/C–O–O–C		O=C–O–		–C–OH/	
	At. Conc. (%)	BE (eV)	At. Conc. (%)	BE (eV)	At. Conc. (%)	BE (eV)
LB	3.75	531.1	10.80	532.2	85.45	533.5
LCNM1	7.44	531.1	45.86	532.3	46.69	533.5
LCNM2	5.85	531.1	24.09	532	70.05	533.5
LCSM1	4.83	531.3	22.45	532.4	72.72	533.6
LCSM2	2.37	531.1	37.92	532.3	59.71	533.5

**Table 5:** The percentages of nitrogen- and sulfur-containing functional groups of materials.

Sample	N 1s						S 2p					
	N1		N2		N3		S1		S2		S3	
	Pyridinic N		Graphitic N		N–H/ Quaternary-N		C–S		C–SO_2_		C–SO_3_	
	At. Conc. (%)	BE (eV)	At. Conc. (%)	BE (eV)	At. Conc. (%)	BE (eV)	At. Conc. (%)	BE (eV)	At. Conc. (%)	BE (eV)	At. Conc. (%)	BE (eV)
LB	-	-	-	-	-	-	57.01	164.0	17.13	166.4	25.86	168.8
LCNM1	62.2	399.8	26.32	400.9	11.48	402.3	38.44	163.6	32.68	166.1	28.88	168.4
LCNM2	77.7	399.8	16.94	401.3	5.36	402.4	41.34	163.8	31.35	166.2	27.32	168.2
LCSM1	-	-	-	-	-	-	40.83	163.9	27.40	166.6	31.77	168.8
LCSM2	-	-	-	-	-	-	21.50	164.0	45.61	166.4	32.89	168.5

**Table 6:** Size, zeta potential and conductivity values for LCMs.

Sample	Size (nm)	Zeta Potential (mV)	Conductivity (mS/cm)
LB	1602.00 ± 96.20	−31.06 ± 0.54	0.029
LCNM1	4.42 ± 0.25	−40.79 ± 0.89	0.103
LCNM2	482.90 ± 44.99	−32.41 ± 0.54	0.029
LCSM1	179.60 ± 3.27	−37.11 ± 0.26	0.119
LCSM2	395.60 ± 26.12	−29.84 ± 1.49	0.032

**Table 7:** DVS data of studied materials.

		BET Data	
Material	^a^ Water Sorption Capacity (% d.b.)	^a^ BET Surface Area (m^2^/g)	^a^ Monolayer (g/g)
LB	11.20	181.741 ± 78.046	0.051
LCNM1	22.40	229.917 ± 41.250	0.065
LCNM2	18.58	139.597 ± 07.819	0.039
LCSM2	42.68	140.973 ± 13.475	0.040
LCSM1	21.17	318.095 ± 39.089	0.090

^a^ Data are expressed as the mean ± standard deviation (n = 3).

**Table 8:** Fluorescence lifetime decay curve fitting parameters for the studied LCMs.

Sample	τ_1_ (ns)	a_1_ (%)	f_1_	τ_2_ (ns)	a_2_ (%)	f_2_	τ_3_ (ns)	a_3_ (%)	f_3_	χ^2^	<τ> (ns)
LCNM1	2.65	41.93	0.24	0.42	18.57	0.02	8.53	39.50	0.74	1.11	6.96
LCNM2	2.47	46.36	0.20	0.49	19.19	0.17	9.90	34.45	0.61	1.00	7.49
LCSM1	2.76	44.93	0.24	0.43	12.55	0.02	8.53	42.52	0.74	1.06	7.02
LCSM2	2.70	32.72	0.28	0.41	41.72	0.05	8.15	25.55	0.66	1.11	6.19

## Data Availability

The data presented in this study are available in the article.
